# Nurse managers' experience with ethical issues in six government hospitals in Malaysia: A cross-sectional study

**DOI:** 10.1186/1472-6939-12-23

**Published:** 2011-11-16

**Authors:** Maizura binti Musa, Md Harun-Or-Rashid, Junichi Sakamoto

**Affiliations:** 1 Young Leaders' Program in Healthcare Administration, Nagoya University Graduate School of Medicine, Nagoya, Japan; 2 Medical Practice Division, Ministry of Health, Putrajaya, Malaysia

## Abstract

**Background:**

Nurse managers have the burden of experiencing frequent ethical issues related to both their managerial and nursing care duties, according to previous international studies. However, no such study was published in Malaysia. The purpose of this study was to explore nurse managers' experience with ethical issues in six government hospitals in Malaysia including learning about the way they dealt with the issues.

**Methods:**

A cross-sectional study was conducted in August-September, 2010 involving 417 (69.2%) of total 603 nurse managers in the six Malaysian government hospitals. Data were collected using three-part self-administered questionnaire. Part I was regarding participants' demographics. Part II was about the frequency and areas of management where ethical issues were experienced, and scoring of the importance of 11 pre-identified ethical issues. Part III asked how they dealt with ethical issues in general; ways to deal with the 11 pre-identified ethical issues, and perceived stress level. Data were analyzed using descriptive statistics, cross-tabulations and Pearson's Chi-square.

**Results:**

A total of 397 (95.2%) participants experienced ethical issues and 47.2% experienced them on weekly to daily basis. Experiencing ethical issues were not associated with areas of practice. Top area of management where ethical issues were encountered was "staff management", but "patient care" related ethical issues were rated as most important. Majority would "discuss with other nurses" in dealing generally with the issues. For pre-identified ethical issues regarding "patient care", "discuss with doctors" was preferred. Only 18.1% referred issues to "ethics committees" and 53.0% to the code of ethics.

**Conclusions:**

Nurse managers, regardless of their areas of practice, frequently experienced ethical issues. For dealing with these, team-approach needs to be emphasized. Proper understanding of the code of ethics is needed to provide basis for reasoning.

## Background

The advances in medicine and a more demanding healthcare environment have given rise to various complex ethical issues. Together, they have caused increasing pressure to healthcare professionals, of which nurses are of no exception. A survey by the American Nurses Association (ANA) Center for Ethics and Human Rights at the ANA Convention in 1994 reported that as high as 79% of their members were confronted with ethical issues on daily to weekly basis [1]. What is meant by "ethical issue" is the dilemma on how someone should act morally in a difficult situation, which is "a question of what is the good or right thing to do" [2]. The first so called "code of ethics" was made by Florence Nightingale in 1893 [3]. Since 1953, however, behaviors that are accepted as ethical in nursing practice are those according to the International Council of Nurses' "ICN Code of Ethics for Nurses", which nowadays becomes the basis for nursing practice standards worldwide. It comprises of matters regarding nurses' relationships with "people", "practice", "profession", and "co-workers" [4].

However, this paper is not about overall nursing ethics, but about ethical issues that affect nurse managers. Our interest in studying their experiences was somewhat due to their unique position. Apart from being front-liners, they also play managerial roles. In other words, nurse managers unlike other nurses are involved not only in patient care, but also have supervision and management responsibilities. They are also members of multi-disciplinary teams and sub-ordinates to doctors [5-7].

Though various ethical issues encountered by nurse managers which warrant attention have been identified, we were unaware of any published study on ethical issues in nursing practice, let alone faced by nurse managers in Malaysia. However, medicine is also advancing here in parallel with rising public expectations to have what they believe as high-quality nursing care. Therefore, it is expected that nurse managers in Malaysia too are experiencing frequent ethical issues. In the absence of previous formal study, the actual burden has not been clearly understood. Moreover, Brosnan J et al. (1997) emphasized that in order to prepare nurse managers for dealing with ethical issues, the first step is to realize what they are, and that such issues are expected to arise in their course of work. The ethical issues may either be rare or day-to-day recurring issues [8].

DeWolf Bosek MS et al. (2006) claimed that understanding nurse managers' role during ethical situation is contingent upon understanding the role and responsibilities associated with the profession [9]. They claimed that nurse managers are the key persons responsible for assisting other nurses evaluate their moral commitments. They also have ethical requirement to lead by example during both easy and desperate times [9]. Marquis BL et al. (2006) also emphasized that nurse managers' responsibilities are beyond that expected from other nurses during an ethical situation [10]. For these reasons, they are expected to be competent in dealing with ethical issues experienced.

We therefore attempted a study on nurse managers in six government hospitals in Malaysia. Its purpose was to explore nurse managers' experience with ethical issues, and to find out how they dealt with the issues. Although we understood that ethical issues are usually dealt with using multi-step approach [11], we were interested in their main action for dealing with the issues.

## Methods

This cross-sectional study was conducted in August and September 2010 involving six Malaysian government hospitals. For convenience, we selected only those major hospitals in Selangor, Putrajaya and the capital city, Kuala Lumpur.

### Study population

All nurse managers of the six hospitals were invited to participate in the study. Two categories were targeted. They were the matrons and sisters occupying managerial and supervisory positions. The matrons are more senior nurses (more than 10 years of experience) and of the highest position. They supervise all nurses including the sisters. The sisters manage the wards and supervise other nurses under them, while they themselves report to the matrons. Apart from this, both are also involved in patient care. We excluded those less than one year of experience as nurse manager.

### Instrument

The study used structured self-administered questionnaire and informed-consent form written in both English and Malaysian language. The questionnaire prepared was guided by the understanding about nursing practice and the code of ethics under Malaysia's nursing practice laws [12]. We used sets of questions with multiple-choice answers provided in order to minimize inconsistencies in responses. Wherever suitable, space was provided to allow participants indicate other answers not listed. A draft version was piloted with five nurse managers. After feedback consideration, final questionnaire comprised of three parts:

Part I: This part is about participants' demographics. These include age-groups, gender, position, years of practice, years of practice as nurse manager, area of practice and hospital name. We also asked whether participants were aware of any committee that discuss ethical issues in the hospitals and their personal participation. Personal identification was not required to ensure anonymity of the participants.

Part II: This part questioned about participants' experiences (whether they have experienced ethical issues since they became nurse managers and if so, how often), areas of management where ethical issues were encountered (five pre-identified areas), and scoring of the importance of 11 pre-identified ethical issues which were important issues faced by nurses and nurse managers based on literature review.[13-15] The issues were: (1) protecting patients' rights; (2) care of patients' quality of life; (3) providing care with possible risks to own health and safety; (4) respecting informed consent by patients to treatment; (5) use of physical or chemical restraints on patients; (6) determining when death occurs; (7) prolonging dying process without appropriate reason; (8) hiding information regarding patients' actual condition from patients or family; (9) working with unethical or incompetent colleague; (10) problems of staffing that limit patients' access to nursing care; and (11) problems of medical resource management that limit patients' access to nursing care. A four-point Likert scale was used for scoring, ranged from 1 (not important) to 4 (very important).

Part III: This part collected responses on how participants generally dealt with ethical issues. Six options were listed, which were: (1) discuss with doctors; (2) discuss with other nurses; (3) refer to code of ethics and other guidelines; (4) refer to the hospital's management team; (5) refer to appointed committee that discuss ethical issues; and (6) try solving the issues alone. We also asked how they dealt with the 11 pre-identified ethical issues in part II by using similar six options listed, and inquired their perceived stress level when experiencing these issues by using a four-point Likert scale from 1 (no stress) to 4 (very stressful).

### Data collection

The questionnaires and informed-consent forms were distributed to 603 nurse managers by the hospitals' Heads of Nursing Department (the head matrons), after explanation provided about the study. Completed questionnaires and informed-consent forms were compiled separately by the head matrons before handed in to the researcher. In total, 430 (71.3%) nurse managers responded but 417 (69.2%) questionnaires were analyzed. Thirteen questionnaires were excluded from the analyses due to incomplete information.

### Statistical analyses

Statistical analyses were conducted using the Statistical Package for Social Sciences (SPSS) 18 software (by SPSS Inc. 233, South Wacker Drive, 11^th ^Floor, Chicago, IL 60606-6412). Descriptive statistics (frequency and percentage) were used to obtain information about participants' experience, participation in committees that discuss ethical issues, options for dealing generally with ethical issues, stress experience, and their main action (from the six options listed) for dealing with the 11 pre-identified ethical issues. For the 11 pre-identified ethical issues, mean importance and mean stress scores were also calculated from the sum of scores given to each issue, divided by total number of participants. Analyses using cross-tabulation and Pearson's Chi-square were done to determine if there were any association between participants' areas of practice with matters of interest, namely participants' experience with ethical issues including frequency of experience, and options chosen for dealing with the issues. Furthermore, we used similar analyses to figure out the relation between participants' participation in committees that discuss ethical issues with the options they chose to deal with the issues. A p value of less than .05 was considered significant.

### Approvals

Approvals were obtained from the head matrons and hospital directors prior to questionnaires distribution. Malaysia's National Institute for Health Behavioral Research approved the overall design and conduct of the study. This study was exempted from the Ministry of Health of Malaysia's Research Ethics Committee's full Board approval.

## Results

### Participants' demographics

In total, 52 matrons and 365 sisters participated in the study and majority (39.3%) were between 51 to 60 years of age. They were predominantly female. The majority have been in practice between 11 to 30 years but most have only been practicing as nurse managers for 5 years or less. Participants were also mostly from the medical care and surgical care areas of practice. Only 72 participants were involved in committees that discuss ethical issues. The details of the participants' demographics are shown in Table 1.

**Table 1:** Participants' demographics

Variables	Frequency	Percentage
**Age-group in years (N = 417)**		
20-30	1	0.2
31-40	96	23.0
41-50	154	36.9
51-60	164	39.3
> 60	2	0.5
**Sex (N = 417)**		
Female	416	99.8
Male	1	0.2
**Position as nurse manager (N = 417)**		
Matron	52	12.5
Sister	365	87.5
**Years of practice (N = 402)**		
≤ 10	10	0.7
11-20	179	38.4
21-30	159	36.2
> 30	54	20.9
**Years of practice as nurse manager (N = 413)**		
≤ 5	337	80.8
6-10	58	13.9
11-15	13	3.1
> 15	5	1.2
**Area of practice (N = 412)**		
Multi-clinical disciplines	40	9.6
Medical care^a^	113	27.1
Surgical care^b^	109	26.1
Obstetrics & Gynecology care	70	16.8
Pediatrics & Pediatric Surgical care	35	8.4
Anesthesiology & Critical care	21	5.0
Non-clinical service	12	2.9
Emergency care	12	2.9
**Participation in committee that discuss ethical issues (N = 273)**^c^		
Yes	72	26.4
No	201	73.6

^a^Medical care includes general medicine, cardiology, nephrology, neurology, psychiatry and rehabilitative care.

^b^Surgical care includes general surgery, orthopedics, urology, otorhinolaryngology, cardiothoracic and neurosurgery.

^c^From 273 nurse managers who knew there were committees that discuss ethical issues in their hospitals.

### Ethical issues experience

There were 397 (95.2%) nurse managers who experienced ethical issues at some point in their practice. Cumulatively, 47.2% participants experienced ethical issues between weekly to daily. Some participants could not delineate exactly the frequency of experience, whether or not it was yearly or monthly, and chose "occasional" as their answer instead. With regards to areas of management, ethical issues mostly experienced were regarding "staff management", followed by "patient care" and "quality of service delivery" (Table 2).

**Table 2:** Experience with ethical issues

Variables	Frequency	Percentage
**How often ethical issues were experienced (N = 417)**		
Never	20	4.8
Occasional	76	18.2
Yearly	49	11.8
Once a month	72	17.3
Few issues per month	3	0.7
Once a week	32	7.7
Few issues per week	89	21.3
Daily	76	18.2
**Areas of management where ethical issues were experienced**^a^		
Staff management	281	67.4
Patient care	271	65.0
Quality of service delivery	264	63.3
Staff competency assurance	178	42.8
Medical resource management	88	21.1

^a^Multiple answers were accepted. The frequency reflects the total number of nurse managers who chose each answer listed in relation to the total number of nurse managers and similarly, the percentage is the percentage of those who gave answers to the questions in relation to the total number of nurse managers.

For the 11 pre-identified ethical issues, the most important issue was "protecting patients' rights". From the mean importance rating order, we also realized that more important ethical issues were in the areas of "patient care". Details are as per Table 3. Of note, we could not find any significant association between participants working in certain areas of practice and their experience with ethical issues.

**Table 3:** The 11 pre-identified ethical issues: mean importance and mean stress scores

**No**.	Ethical issues	Mean importance **score**^a^	Mean importance rating	Mean stress **score**^b^	Mean stress rating
					
1.	Protecting patients' rights	3.82	1	1.73	11
2.	Care of patients' quality of life	3.79	2	1.84	10
3.	Providing care with possible risks to own health and safety	3.77	3	2.37	5^c^
4.	Respecting informed consent by patients to treatment	3.57	4	1.94	9
5.	Use of physical or chemical restraints on patients	2.78	7	2.07	8
6.	Determining when death occurs	2.35	9	2.19	7
7.	Prolonging dying process without appropriate reason	2.04	11	2.37	5^c^
8.	Hiding information regarding patients' actual condition from patients or family	2.28	10	2.43	4
9.	Working with unethical or incompetent colleague	2.44	8	3.10	1
10.	Problems of staffing that limit patients' access to nursing care	3.18	5	2.87	2
11.	Problems of medical resource management that limit patients' access to nursing care	3.13	6	2.77	3

^a^Based on scale of 1 to 4: 1 = not important, 2 = slightly important, 3 = important, 4 = very important

^b^Based on scale of 1 to 4: 1 = not stressful, 2 = slightly stressful, 3 = stressful, 4 = very stressful

^c^Same rank because of equal mean

### Dealing with ethical issues

Altogether, 249 participants felt stressful when dealing with ethical issues and those who didn't was 168. This difference was statistically significant (p < .001). This means, more than half (59.7%) reported they felt stressful. From the 11 pre-identified ethical issues, top most stressful issue was "working with unethical or incompetent colleague". However, ethical issues regarding "patient care" were relatively less stressful (Table 3).

For options in dealing with ethical issues, majority (80.8%) participants chose "discuss with other nurses" compared to 52.5% who chose "discuss with doctors" as one of the options. Lesser participants chose "refer to code of ethics and other guidelines'" and "refer to appointed committee that discuss ethical issues" (Table 4). However, for the 11 pre-identified ethical issues many thought that "discuss with doctors" was the main action, especially those related to "patient care" (Table 5).

**Table 4:** Dealing with ethical issues in general

**Options in dealing with ethical issues**^a^	Frequency	Percentage
Discuss with doctors	219	52.5
Discuss with other nurses	337	80.8
Refer to code of ethics or other guidelines	221	53.0
Refer to the hospital's management team	298	71.5
Refer to appointed committee that discuss ethical issues	78	18.7
Try solving the issues alone	53	12.7
Other methods	0	0.0

^a^Multiple answers were accepted. The frequency reflects the total number of nurse managers who chose each answer listed in relation to the total number of nurse managers. Similarly, the percentage is the percentage of those who gave answers to the questions in relation to the total number of nurse managers.

**Table 5:** Participants' main actions in dealing with the 11 pre-identified ethical issues

	Ethical issues	Main action	**Percentage of participants**^a^
1.	Protecting patients' rights	Discuss with doctors	45.0
2.	Care of patients' quality of life	Discuss with doctors	44.1
3.	Providing care with possible risk to own health and safety	Discuss with doctors	42.4
4.	Respecting informed consent by patients to treatment	Discuss with doctors	66.2
5.	Use of physical or chemical restraints on patients	Discuss with doctors	65.4
6.	Determining when death occurs	Discuss with doctors	79.8
7.	Prolonging dying process without appropriate reason	Discuss with doctors	79.3
8.	Hiding information regarding patients' actual condition from patients or family	Discuss with doctors	67.7
9.	Working with unethical or incompetent colleague	Discuss with other nurses	34.3
10.	Problems of staffing that limit patients' access to nursing care	Refer to the hospital's management team	40.9
11.	Problems of medical resource management that limit patients' access to nursing care	Refer to the hospital's management team	40.3

^a^Percentage of participants that selected the main action

From 273 participants who knew their hospitals have committees that discuss ethical issues, 208 (76.2%) did not choose "refer to appointed committee that discuss ethical issues" as one of the ways to deal with the issues (x² = 13.546, d.f. = 1, p < .001). Moreover, from 72 participants who claimed they were involved in such committees, 66.7% did not choose "refer to appointed committee that discuss ethical issues" (x² = 4.89, d.f. = 1, p = .027). We found no association between practicing in certain areas of practice and the options chosen for dealing with ethical issues.

## Discussion

This study provides probably the first empirical data and responses to ethical issues by nurse managers in Malaysian hospitals. With this, the burden of ethical issues for nurse managers and the choice of ways for dealing with them could be understood.

### Ethical issues experience

The burden of experience is notably high, where majority nurse managers had previous experiences and almost half of total participants experienced ethical issues between weekly to daily basis. Though most participants experienced ethical issues related to "staff management", the fact that "patient care" came second to this shows that nurses at managerial positions still continue to face frequent ethical issues challenges in "patient care". From the 11 pre-identified ethical issues, it is also evident that four of the five most important ethical issues were related to "patient care", which is in agreement with previous studies [13-15]. However, we did not obtain similar result to those studies that identified problems of medical resources allocation as one of the top ethical issues. Another study has also reported that ethical issue of scanty resources' distribution occurred frequently needing difficult decisions to be made on equity of patient care [16]. A logical answer to our different result is because our participants were working under the instruction of the Heads of Department (usually a medical doctor) of each area of practice. This means nurse managers are able to manage resources, but are not solely responsible for decisions on resources allocation, hence not much burdened by this issue.

We found no significant association between areas of practice and experience with ethical issues. This is in line with Wood M's study (1999) which concluded that nurse managers acquired competency in ethics through education and years of experience. Their focus on the types of ethical issues and decision-making were not confined within certain areas of practice [17].

### Dealing with ethical issues

The finding of more than half participants reported feeling stressful suggests that dealing with ethical issues is rather burdensome. Among the 11 pre-identified ethical issues, "working with unethical or incompetent colleague" was voted the most stressful. Despite a decline in quality due to overproduction of nurses, majority still possess high competency and professionalism [18]. Therefore, unfamiliar situation like dealing with unethical or incompetent colleague would be considerably stressful. Issues regarding "patient care" were relatively less stressful than other areas of management more likely because nurse managers tend to discuss these with doctors and not just among themselves.

Majority participants generally chose "discuss with other nurses" to deal with ethical issues. For sensitive issues regarding "patient care", nurse managers felt "discuss with doctors" was the best option probably as the immediate reliable measure for dealing with the issues. Filipova AA's study (2009) suggested that the way ethical issues are handled reflects the ethics of an organization [19]. Hence, this result implies that the working environment between doctors and nurse managers are still at best situation in the hospitals.

Our study also found out that those who participated in committees that discuss ethical issues ("ethics committees") did not prefer referring the issues to the committee. A logical explanation would be the understanding that "ethics committees" approach depends on the involvement of many parties from different backgrounds [20]. Hence, referring all issues to "ethics committees" would not be feasible due to logistic reasons. If issues are small and require decisions be made quickly, it is understandable that one would utilize a more convenient and immediate measure instead. In addition, de Casterle BD et al. (2002) also pointed out that past discussions leading nowhere or ending in negative consequences could create an atmosphere in which frequent ethical issues are not discussed [21]. Conversely, Parker F (2007) claimed that "ethics committees" meetings could promote professional growth by providing them the opportunity for learning how to address ethical issues from multi-disciplinary experts' different perspectives [22]. Hence, nurse managers engaged in "ethics committees" could have learnt from previous decisions on how to address some ethical issues.

To date, the Malaysia Nursing Board has come out not only with its code of ethics, but with 25 other guidelines to ensure nursing practice is carried out according to the accepted norms. Although this is so, only about half of total nurse managers would refer to the code of ethics for dealing with ethical issues. Nevertheless, similar finding was also obtained in other studies. When deciding on how to handle ethical issues, nurse managers usually rely on three things: their personal skills, the management team or other nurses, or the code of ethics. In most cases, the latter was the least referred. Its usefulness in decision making had even been criticized. One criticism was that although code of ethics compliance is crucial to prevent unethical or illegal practices, it inadequately guides one's thoughts in formulating a viewpoint or judgement in dealing with ethical issues [23,24].

The fact that we could not find any association between areas of practice and choice of dealing with ethical issues points out to the suggestion that any organizational improvement going to be made need not be targeted to certain areas of practice.

A couple of study limitations related to the use of self-administered questionnaire are worthy of attention. Firstly, we used a list of pre-identified ethical issues originally intended to avoid inconsistencies in the answers. Hence, we did not ask their experience with issues such as ethics of organ transplant and surrogate decision-making as in a previous study [14]. However, some of our hospitals have no organ transplant service, so participants were unable to answer it anyway; moreover, surrogacy is prohibited in the country. In addition, not using open-ended questions or exhaustive list in this study lead to limited options that precluded new input and provision of exact descriptions from the participants on the types of ethical issues they experienced. As a result, we could not identify whether or not there were any ethical issues that may be unique to nurse managers in these hospitals especially those that may be related to their cultural and social backgrounds. Secondly, our participants were not randomly selected. While questionnaires were distributed to all nurse managers with the expectation of full participation, only two-third responded. Therefore, possibility exist that non-respondent nurse managers might be representative of specific group of population with unique experience and understanding of ethical issues. However, our method obtained better response compared to 15% - 52% in previous studies which utilized traditional mailing system as communication means [13-15], where responses were largely dependent upon participants' willingness to return pre-stamped envelopes back to the sender. A third limitation is that participants' experiences were based on their recall of past encounters of ethical issues. We solely relied on their own knowledge about ethical issues to correctly recall they have previously experienced them.

## Conclusions

The frequent experience with ethical issues suggests that awareness is still needed for nurses who already reached nurse manager positions. Ethical issues require reasoned and rational approach to be dealt with appropriately. Nurse managers regard support from other nurses as essential but discussion with co-workers such as doctors is still important especially when dealing with "patient care" ethical issues. This points out to the consideration that team-approach is necessary, ideally the team which can serve as a quick and reliable resource for dealing with the issues, and this is best combined with proper understanding of the code of ethics as basis for reasoning. We hope this information can provide basis for enhancing support for nurse managers' workforce and professional developments, and to improve organizational approach in dealing with ethical issues. We recommend further studies, including qualitative studies to be conducted in hospitals nationwide, in other types of healthcare institutions, or among nurses of different levels in order to obtain wider perspectives.

## Competing interests

The authors declare that they have no competing interests.

## Authors' contributions

MbM conducted the research, did the statistical analyses and wrote the initial manuscript, MHOR and JS reviewed the statistical analyses and edited the manuscript. All the authors have read and approved the manuscript.

## Pre-publication history

The pre-publication history for this paper can be accessed here:

http://www.biomedcentral.com/1472-6939/12/23/prepub

## References

[B1] Scanlon C Survey yields significant results Communique 1994 4 1 3

[B2] Redman BK Fry ST Nurses' ethical conflicts: what is really known about them? Nurs Ethics 2000 7 360 366 11221412 10.1177/096973300000700409

[B3] Bostridge M Florence Nightingale: The Making of an Icon 2008 Illustrated edn: Indiana University Press

[B4] International Council of Nurses The ICN Code of Ethics for Nurses 2005 Geneva, Switzerland: Imprimerie Fornara

[B5] Hisar F Karadag A Determining the professional behaviour of nurse executives Int J Nurs Pract 2010 16 335 341 10.1111/j.1440-172X.2010.01849.x 20649664

[B6] Wilson-Barnett J Ethical dilemmas in nursing J Med Ethics 1986 12 123 126 135 10.1136/jme.12.3.123 3761331 PMC1375348

[B7] Mitchell JR Clin Res Is nursing any business of doctors? A simple guide to the "nursing process" Br Med J 1984 288 216 219 10.1136/bmj.288.6412.216 PMC1444455 6419863

[B8] Brosnan J Roper JM The reality of political ethical conflicts. Nurse manager dilemmas J Nurs Adm 1997 27 42 46 9300014 10.1097/00005110-199709000-00010

[B9] DeWolf Bosek MS Stammer K Ethical commitments during desperate times JONAS Healthc Law Ethics Regul 2006 8 123 128 10.1097/00128488-200610000-00007 17149041

[B10] Marquis BL Huston CJ Leadership Roles and Management Functions in Nursing: Theory and Application 2008 6 Philadelphia PA 19106: Lippincott Williams & Wilkins

[B11] Toren O Wagner N Applying an ethical decision-making tool to a nurse management dilemma Nurs Ethics 2010 17 393 402 10.1177/0969733009355106 20444780

[B12] Nurses Act 1950 (Revised 1969) & Nurses Registration Regulations 1985 http://www.agc.gov.my/Akta/Vol.%201/Act%2014.pdf

[B13] Cooper RW Frank GL Hansen MM Gouty CA Key ethical issues encountered in healthcare organizations: the perceptions of staff nurses and nurse leaders J Nurs Adm 2004 34 149 156 10.1097/00005110-200403000-00008 15024242

[B14] Ulrich CM Taylor C Soeken K O'Donnell P Farrar A Danis M Grady C Everyday ethics: ethical issues and stress in nursing practice J Adv Nurs 2010 66 2510 2519 10.1111/j.1365-2648.2010.05425.x 20735502 PMC3865804

[B15] Aitamaa E Leino-Kilpi H Puukka P Suhonen R Ethical problems in nursing management: the role of codes of ethics Nurs Ethics 2010 17 469 482 10.1177/0969733010364896 20610580

[B16] Williams A Thinking about equity in health care J Nurs Manag 2005 13 397 402 10.1111/j.1365-2834.2005.00578.x 16108777

[B17] Woods M A nursing ethic: the moral voice of experienced nurses Nurs Ethics 1999 6 423 433 10696189 10.1177/096973309900600508

[B18] Nemie JKP Challenges for the nursing profession in Malaysia: Evolving legal and ethical standards Journal of Nursing Law 2009 13 54 62 10.1891/1073-7472.13.2.54

[B19] Filipova AA Licensed nurses' perceptions of ethical climates in skilled nursing facilities Nurs Ethics 2009 16 574 588 10.1177/0969733009106650 19671644

[B20] Keffer MJ Why nursing ethics committees? HEC Forum 1997 9 50 54 10.1023/A:1008869429968 10166912

[B21] Dierckx de Casterle B Meulenbergs T van de Vijver L Tanghe A Gastmans C Ethics meetings in support of good nursing care: some practice-based thoughts Nurs Ethics 2002 9 612 622 10.1191/0969733002ne555oa 12449998

[B22] Parker FM Ethics: The power of one OJIN: Online Journal of Issues in Nursing 2007 13

[B23] Borawski DB Resources used by nurse administrators in ethical decision-making J Nurs Adm 1994 24 17 22 8133319

[B24] Pattison S Are nursing codes of practice ethical? Nurs Ethics 2001 8 5 18 16010905 10.1177/096973300100800103

